# Metabolomics biomarkers and related pathways in acute renal injury: a bibliometric investigation, meta-analysis, and systematic review

**DOI:** 10.3389/fmed.2025.1679349

**Published:** 2025-10-20

**Authors:** Zixiang Kai, Keming Yun, Yanbing Su, Junhu Li, Yuxiang Liu

**Affiliations:** ^1^School of Forensic Medicine, Shanxi Medical University, Jinzhong, China; ^2^Office of Research, Shanxi Bethune Hospital, Shanxi Academy of Medical Sciences, Tongji Shanxi Hospital, Third Hospital of Shanxi Medical University, Taiyuan, China; ^3^Shanxi Provincial Key Laboratory of Kidney Disease, Department of Nephrology, Shanxi Provincial People's Hospital (The Fifth Clinical Medical College of Shanxi Medical University), Taiyuan, China

**Keywords:** acute kidney injury, metabolomics, biomarker, bibliometric investigation, meta-analysis

## Abstract

**Objective:**

Early diagnosis of Acute Kidney Injury (AKI) is critical for improving patient outcomes. This study aims to provide a comprehensive overview of the research landscape and to identify key metabolic biomarkers and pathways associated with AKI through bibliometric analysis and meta-analysis.

**Methods:**

We conducted a bibliometric analysis of scientific articles on metabolomics in AKI published between 2005 and 2025. Building on this bibliometric foundation, we performed a systematic review and meta-analysis in accordance with Preferred Reporting Items for Systematic Reviews and Meta-Analyses (PRISMA) guidelines to delve deeper into the synthesis of findings on diagnostic metabolites.

**Results:**

(1) Bibliometric analysis shows rapid growth and international collaboration in AKI metabolomics research. Studies focused on risk factors, management, and underlying mechanisms. (2) Meta-analysis reveals that decreased glycine, lysine, and cystine, along with increased tryptophan, distinguish AKI patients from healthy controls. These metabolites are sensitive diagnostic biomarkers. (3) Enriched pathways identified by Human Metabolome Database (HMDB) and Kyoto Encyclopedia of Genes and Genomes (KEGG) suggest that amino acid metabolism are key contributors to AKI pathogenesis.

**Discussion:**

This systematic review highlights the significant diagnostic and mechanistic value of metabolomics in AKI. The identified metabolite panel and associated pathways offer promising targets for early diagnosis and elucidate critical aspects of the disease’s underlying biology.

## 1 Introduction

Acute kidney injury (AKI) is a common, complex disease with significant clinical variation (1). Studies in Canada and elsewhere show AKI incidence has risen to 1.14–1.62%. Yearly mortality rates range from 27.8 to 32.8% (2). This increase is linked to AKI’s rapid onset. Without early diagnosis and proper treatment, renal function can worsen and lead to multi-organ failure and death (3, 4).

AKI develops through complex, multifactorial mechanisms (5–7). Early and accurate diagnosis, plus timely intervention, is vital to halt or reverse disease progression (6). Traditional diagnostic markers, such as serum creatinine and urine output, often lack real-time sensitivity and may delay assessment (8). New approaches, particularly metabolomics, provide opportunities to elucidate AKI mechanisms and identify reliable early biomarkers (9, 10).

To address this diagnostic challenge, our study maps AKI research activity using bibliometric methods (11). We then systematically review and meta-analyze published studies on metabolomics in AKI (12). This structured process helps identify and validate relevant biomarkers and their associated metabolic pathways.

## 2 Materials and methods

The primary data for this study were derived from previously published research, all of which had already obtained ethical approval from their respective ethics committees. Therefore, the current study is considered exempt from additional ethical review.

### 2.1 Bibliometric analysis

#### 2.1.1 Database selection and search strategy

With the assistance of an information specialist, we searched the Web of Science (2005–2025) for studies on AKI and metabolomics, utilizing comprehensive search terms. No language or other restrictions were applied. References of selected articles were checked, and experts were consulted for additional studies. After excluding unrelated articles, we extracted data (title, country, institution, first author, publication year, corresponding author, journal, citation count, publication type), assigning affiliation/country by the corresponding author’s institution.

#### 2.1.2 Inclusion criteria

The inclusion criteria were refined for data-driven studies and relevance. Studies were included if they met these three conditions: (1) Article Type: “ar” (articles); (2) Publication stage: “final”; (3) Language: English.

#### 2.1.3 Data extraction and quality assessment

We extracted the following information from each study using a standardized data extraction form:

(1) General information: first author, corresponding author, the affiliated country and organization, year of publication, and journal.
(2) Study design, subject Information, and keywords.

To ensure consistency, two team members independently piloted data extraction on 50 random documents. Results were discussed to align the criteria. During extraction, two researchers independently collected data, discussing discrepancies, and a third researcher resolved any unresolved differences.

#### 2.1.4 Data analysis

We used CiteSpace (v5.7. R5W) for bibliometric analysis (13, 14), including visualization and network analysis of author, institution, and keyword relationships. Bibliometric indicators (e.g., publication/citation counts, co-authorship stats) and graphical representations (bar charts, network maps) were generated to provide insights into publication trends, collaboration, and research directions in AKI (13, 15, 16).

### 2.2 Meta-analysis and systematic review

#### 2.2.1 Database selection and search strategy

This review was conducted by the Preferred Reporting Items for Systematic Reviews and Meta-Analyses (PRISMA) guidelines (17). We conducted a comprehensive search of PubMed, Ovid, Cochrane, and Web of Science databases to identify relevant studies published online. We also searched two additional preprint repositories (ESAY and medRxiv). The search strategy utilized the subject termsearch terms “metabolomics,” “lipidomics,” “acute kidney injury,” and their variants.

#### 2.2.2 Study selection and eligibility criteria

We used EndNote X9 (Stanford University, United States) to remove duplicate records. Two researchers independently screened article titles, abstracts, and full texts. A third researcher resolved disagreements regarding selection or inclusion. Studies were included if they met all of the following:

(1) Human studies on AKI using case–control, cross-sectional, or cohort designs;
(2) Comparisons between AKI and non-AKI samples;
(3) Use of high-throughput metabolomics technologies (such as gas chromatography, liquid chromatography (LC), mass spectrometry (MS), or combinations) for metabolite identification in biological fluids or tissue samples;
(4) Analysis of human biospecimens collected prior to surgery.

Studies were excluded if they:

(1) Involved animal models or pregnant women;
(2) were non-original research (reviews, commentaries, editorials, or letters) or duplicate publications;
(3) did not use metabolomics methods to assess metabolite changes;
(4) focused on intracellular metabolite profiling;
(5) were from grey literature lacking sufficient information.

#### 2.2.3 Data extraction and quality assessment

We extracted the following information from each study using a standardized data extraction form:

(1) General information: first author, year of publication, journal;
(2) Demographic information: participant location, age, sample size, biospecimen type, control type;
(3) Methodological information: detection methods used;
(4) Outcome measures: analytical data including means and standard deviations (SD), or odds ratios (OR) and 95% confidence intervals (CI) for association trends;
(5) Study design.

Two researchers independently extracted data; disagreements were resolved with a third researcher. Study quality was assessed using the 9-point Newcastle-Ottawa Scale (NOS) (18), which reviews population selection, comparability, and exposure or outcome. Studies scoring ≥6 were considered moderate to high quality.

#### 2.2.4 Data synthesis and meta-analysis

Following data extraction and assessment, we synthesized the results and performed meta-analysis as described below.

We used R software (version 4.2.1) to perform meta-analyses on biomarkers and metabolic pathways with available metabolite data. Studies with insufficient data or low-quality scores were excluded from the meta-analysis. The original literature in this study employed various metabolite detection techniques, primarily liquid chromatography–tandem mass spectrometry (LC–MS/MS). To integrate data from different platforms and conduct pathway analysis, we focused on the directional changes—upregulation or downregulation—of metabolites in the disease group compared to controls, rather than on absolute concentration values. We identified principal metabolic pathways associated with reported metabolites using the Human Metabolome Database (HMDB) and Kyoto Encyclopedia of Genes and Genomes (KEGG) (19). We used the standardized mean difference (SMD) to estimate the effect size. For studies reporting means, SD, and sample sizes, SMD were calculated using Cohen’s d method. For studies reporting only OR and 95% CI, the Hasselblad and Hedges method was used to convert these into SMD and their standard errors (SE) before inclusion.

In studies with multiple control groups, we selected the control group most age-matched to the AKI group. When multiple analytical methods were employed, we prioritized results obtained using commercial kits over those obtained through internal analytical methods. Heterogeneity among studies was assessed using Cochran’s Q test and *I*^2^ statistics. Significant heterogeneity was defined as *I*^2^ > 50% and *p*-value ≤ 0.10 (20, 21). For results with significant heterogeneity (*I*^2^ > 50%), we performed pooled analyses using the random-effects model. Furthermore, we also specified subgroup analyses to explore potential sources of heterogeneity based on the following factors: patient age (<60 years vs. ≥60 years). Differences between subgroups were formally tested using Cochran’s Q statistic for subgroup differences, with a *p*-value < 0.05 considered statistically significant.

## 3 Results

### 3.1 General trends in metabolomics of AKI research

**Figure 1:**
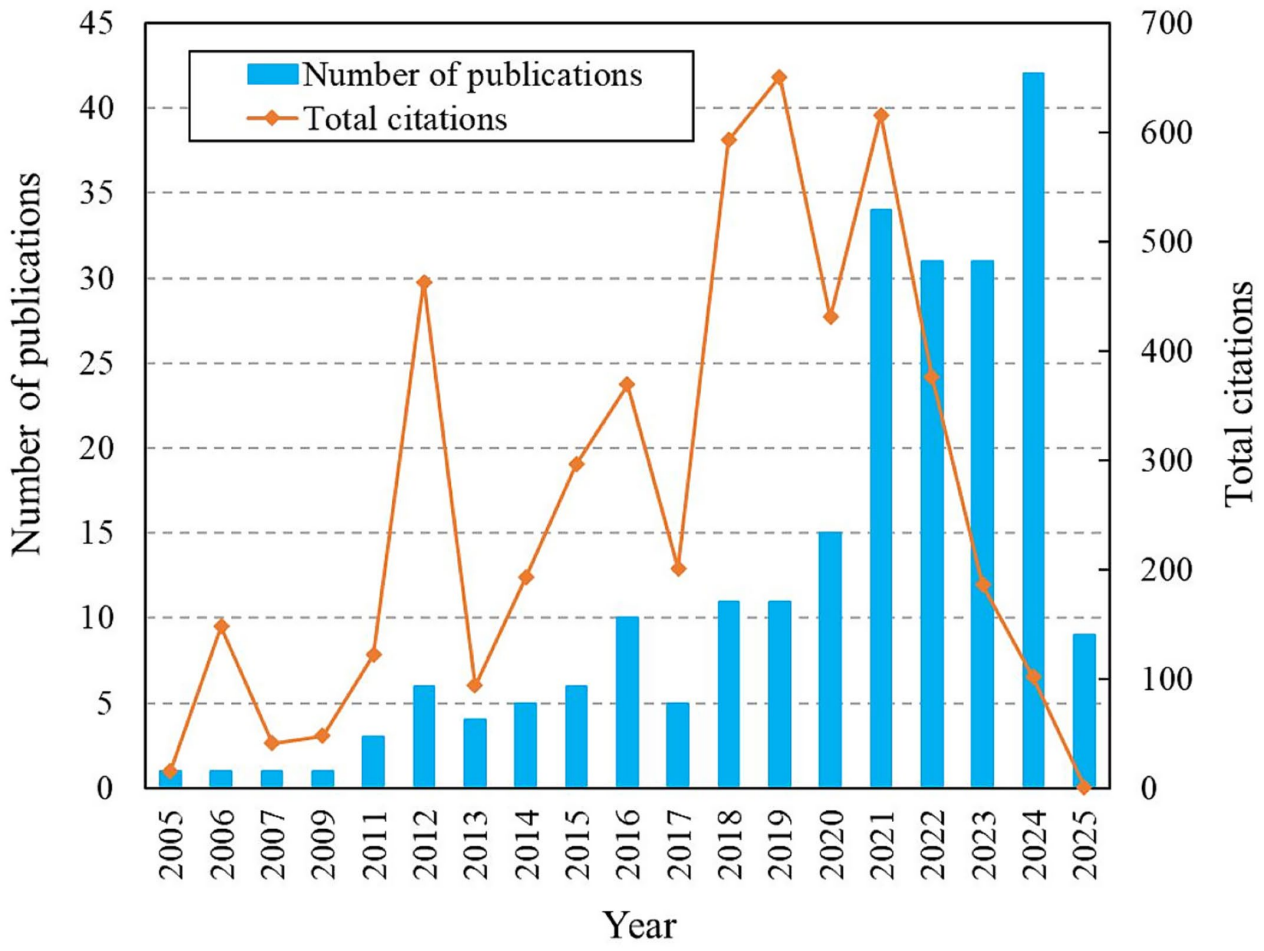
The annual distribution of publications and total citations.

A total of 227 studies have been published in the field of metabolomics in AKI. Figure 1 presents the publication trend and total citation frequency. Research in this area began in 2005, with a slow increase in publications from 2005 to 2010, followed by a gradual rise after 2011. The number of studies increased sharply to 15 in 2020, peaked at 34 in 2021, declined to 31 in 2022, remained stable in 2023, and rose again to 42 in 2024 before decreasing to 9 in 2025. The rapid development and adoption of multi-omics technologies, including metabolomics, contributed to this accelerated growth. The increasing proportion of highly cited papers indicates ongoing improvements in research quality.

**Figure 2:**
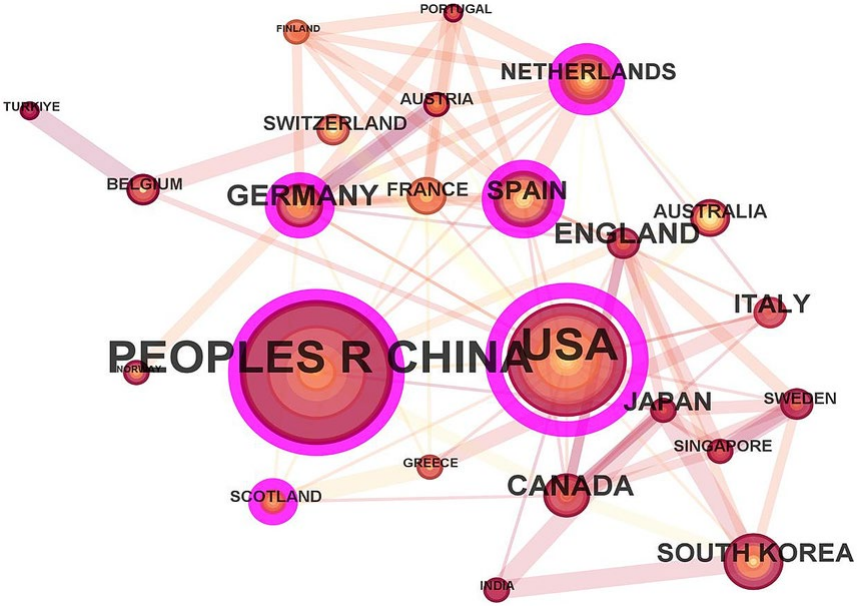
The scientific cooperation network among countries.

From the perspective of international collaboration networks (Figure 2), the United States acts as a central hub, frequently collaborating across regions with China and Italy. European countries, such as Germany, France, Spain, and the Netherlands, form a close-knit group. Although China has a relatively large number of publications, its cross-border cooperation is relatively less. This reflects the coexistence of globalization and regionalization in the field of metabolomics research related to AKI.

**Figure 3:**
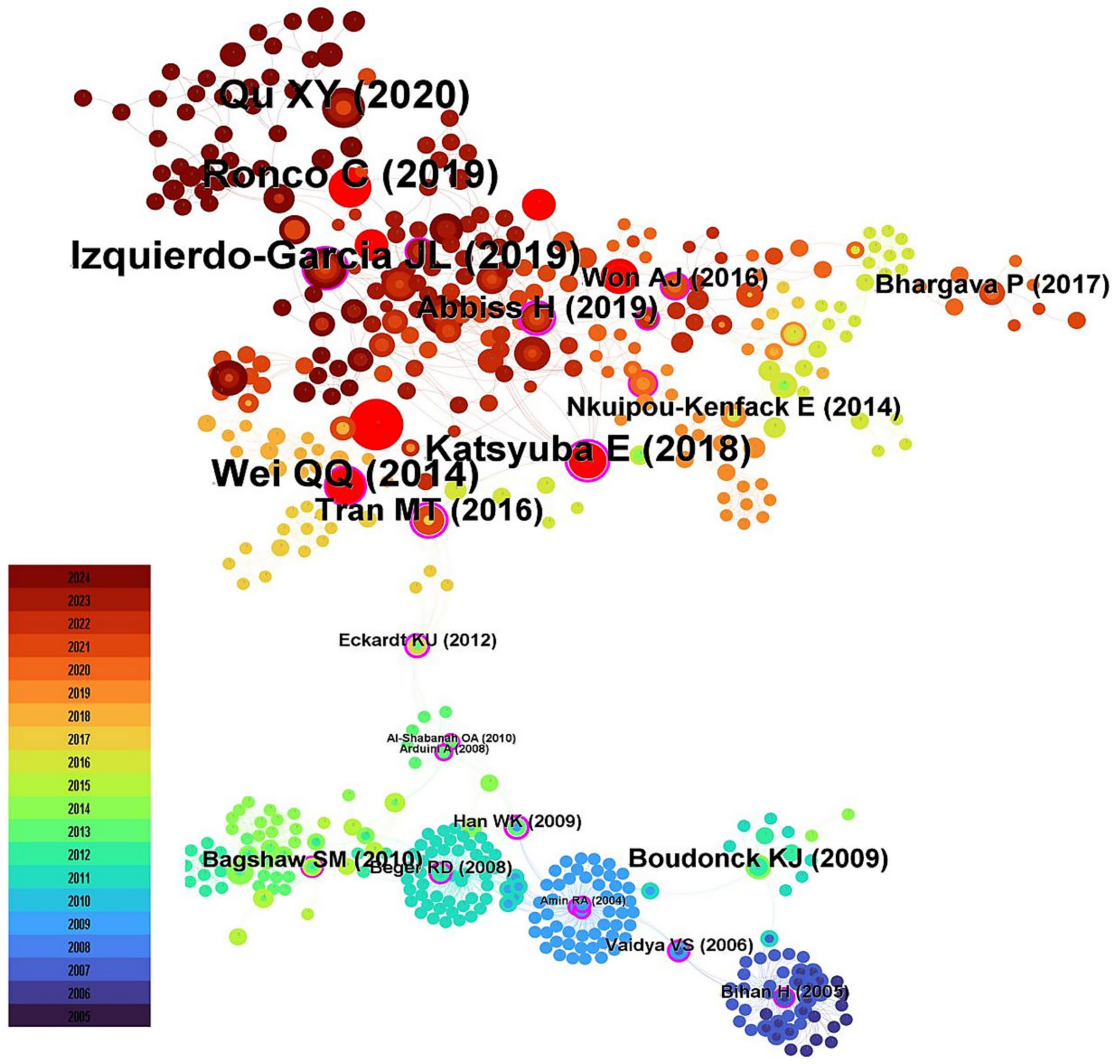
The citation co-occurrence network. The color bar from bottom (blue) to top (red) indicates the year from 2005 to 2025.

**Figure 4:**
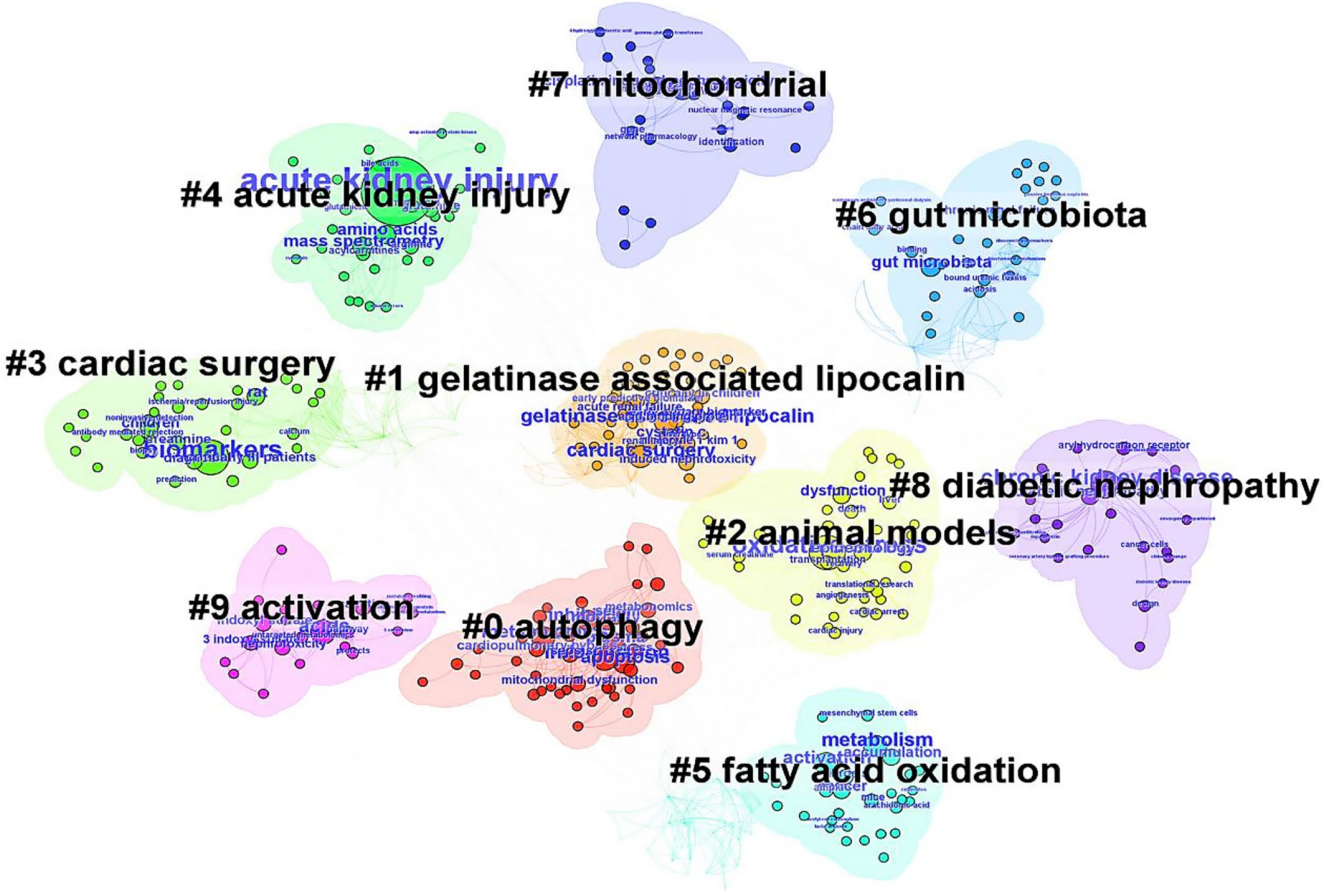
The keyword clusters network in metabolomics of AKI from 2005 to 2025.

The co-citation situation of the literature was analyzed (Figure 3). The study with the highest number of citations was “De novo NAD+ biosynthetic impairment in acute kidney injury in humans” published by Mehr et al. in Nature Medicine in 2018, which was cited 19 times. Among the top 10 most co-cited articles, only 1 was published in 2014, while the remaining 9 were published between 2017 and 2020. The first authors of the 1-Keyword clusters represent the core themes of the research. A keyword co-occurrence analysis identified 343 terms, with 35 appearing at least five times and 14 appearing ten times or more. Notably, oxidative stress and inflammation emerged as prominent keywords. Visualization of keyword co-occurrence (Figure 4) highlights frequently co-occurring terms, revealing research hotspots in AKI metabolomics. Autophagy and gelatinase-associated lipocalin are also significant co-occurring keywords. These findings indicate an increasing research focus on the intrinsic mechanisms of AKI and its potential clinical applications.

### 3.2 Literature search results for the meta-analysis

**Figure 5:**
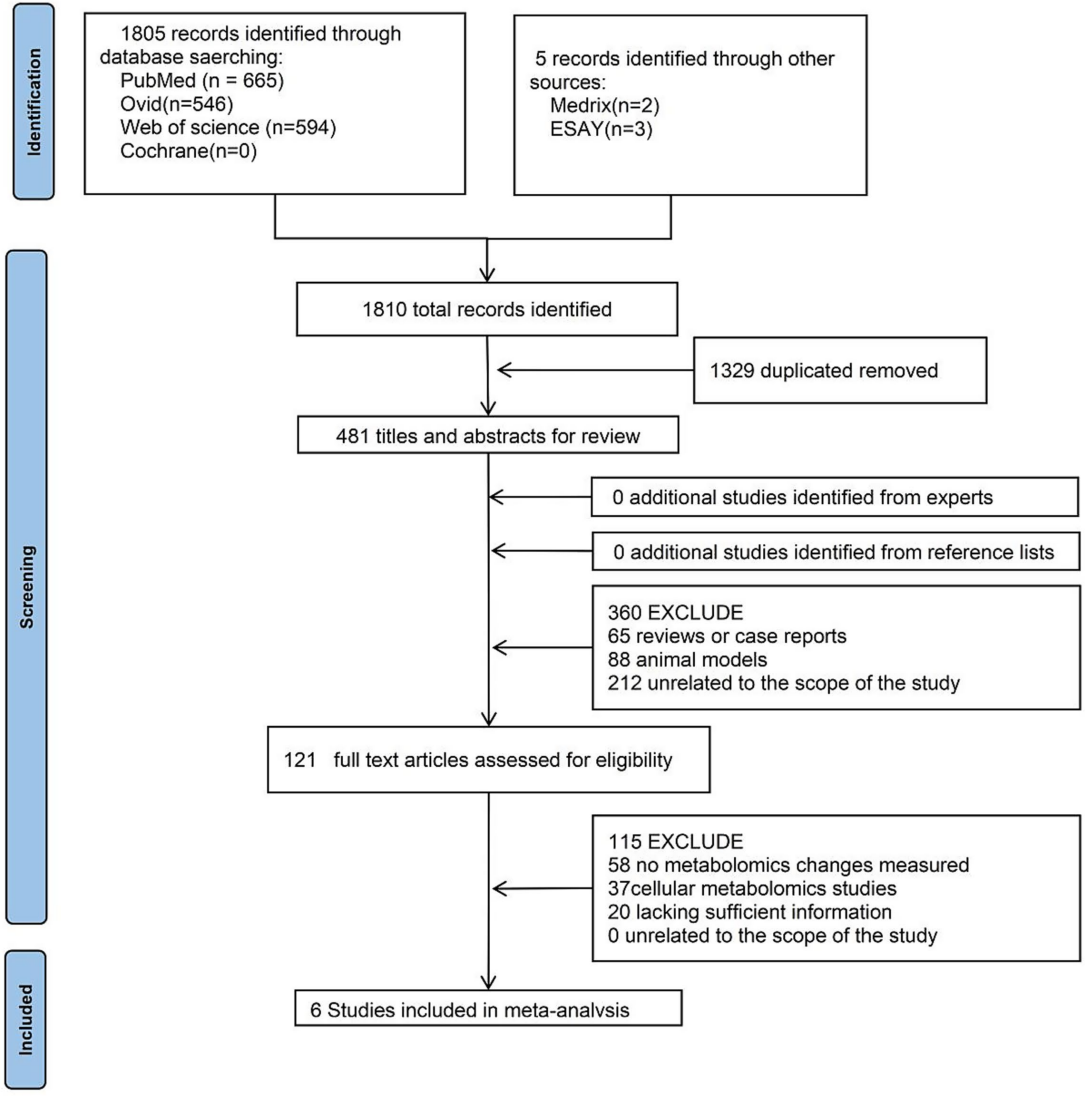
Flowchart of meta-analysis.

Figure 5 shows the literature search and study selection process. An initial database search yielded 1,810 articles. After removing duplicates, 481 articles remained, and their titles and abstracts were independently screened by two reviewers, leading to the exclusion of 360 articles. Manual reference list screening and expert consultation did not identify any additional relevant studies. The full texts of the remaining 121 articles were reviewed, of which 115 were excluded. Ultimately, six studies with extractable data were included in the systematic review.

### 3.3 Characteristics and quality assessment of included studies

Six studies were included and summarized in Table 1 based on extractable data. These studies were published between 2016 and 2024, with sample sizes ranging from 44 to 159 participants (median: 80). All six employed untargeted metabolomics analyses. The geographic distribution included two European studies, three from Asia, and one from North America. Four studies analyzed blood samples, while two analyzed urine samples. These six studies, in terms of both their publication time and sources, align with the annual and regional trends identified through our bibliometric analysis. This provides indirect confirmation of the research trends in bibliometrics and also indicates that the meta-analysis data are representative.

**Table 1:** Six studies included in the meta-analysis.

First author	Publication date	Journal (JCR classification)	Participant location	Age	Sample size (number of AKI)	Biological sample type	Control type	Detection method
Sammy Elmariah, MD (67)	2016	Journal of the American Heart Association (Q1)	Massachusetts General Hospital (United States)	81.90 ± 8.50	44 (9)	Plasma	Case–control	LC–MS
Feng Zhang (71)	2018	Science Reports (Q2)	Shanghai Changzheng Hospital (CHN)	32.71 ± 7.80	66 (30)	Plasma	Case–control	LC–MS/MS
Hyun-Seung Lee (72)	2021	Antioxidants (Q1)	Samsung Medical Center, School of Medicine, Singkyunwan University, South Korea (KOR)	60.20 ± 13.10	97 (28)	Serum	Case–control	LC–MS/MS
Meice Tian (73)	2021	Journal of Thoracic and Cardiovascular Surgery (Q1)	Fuwai Hospital, Chinese Academy of Medical Sciences (CHN)	64 (59–67)	159 (55)	Urine	Cohort studies	LC–MS
Sevilay Erdogan Kablan (68)	2024	Rapid Communications in Mass Spectrometry (Q4)	Ankara City Hospital, Turkey (TR)	–	107 (9)	Plasma	Case–control	LC–MS
Claudia Muhle-Goll (74)	2020	International Journal of Molecular Science (Q1)	Heidelberg Children’s Hospital, Germany (GER)	3.90 (0 ~ 18)	65 (53)	Urine	Case–control	LC–MS

“–” Age was not reported in the article; Age (age range); age ± standard deviation.

The Newcastle-Ottawa Scale (NOS) was used to assess study quality, with scores ranging from 6 to 6.5. The mean score was 6.38 ± 1.1 (mean ± SD), and all six studies were considered to be of moderate to high quality (score ≥ 6).

### 3.4 Meta-analysis of biomarker expression in AKI

**Figure 6:**
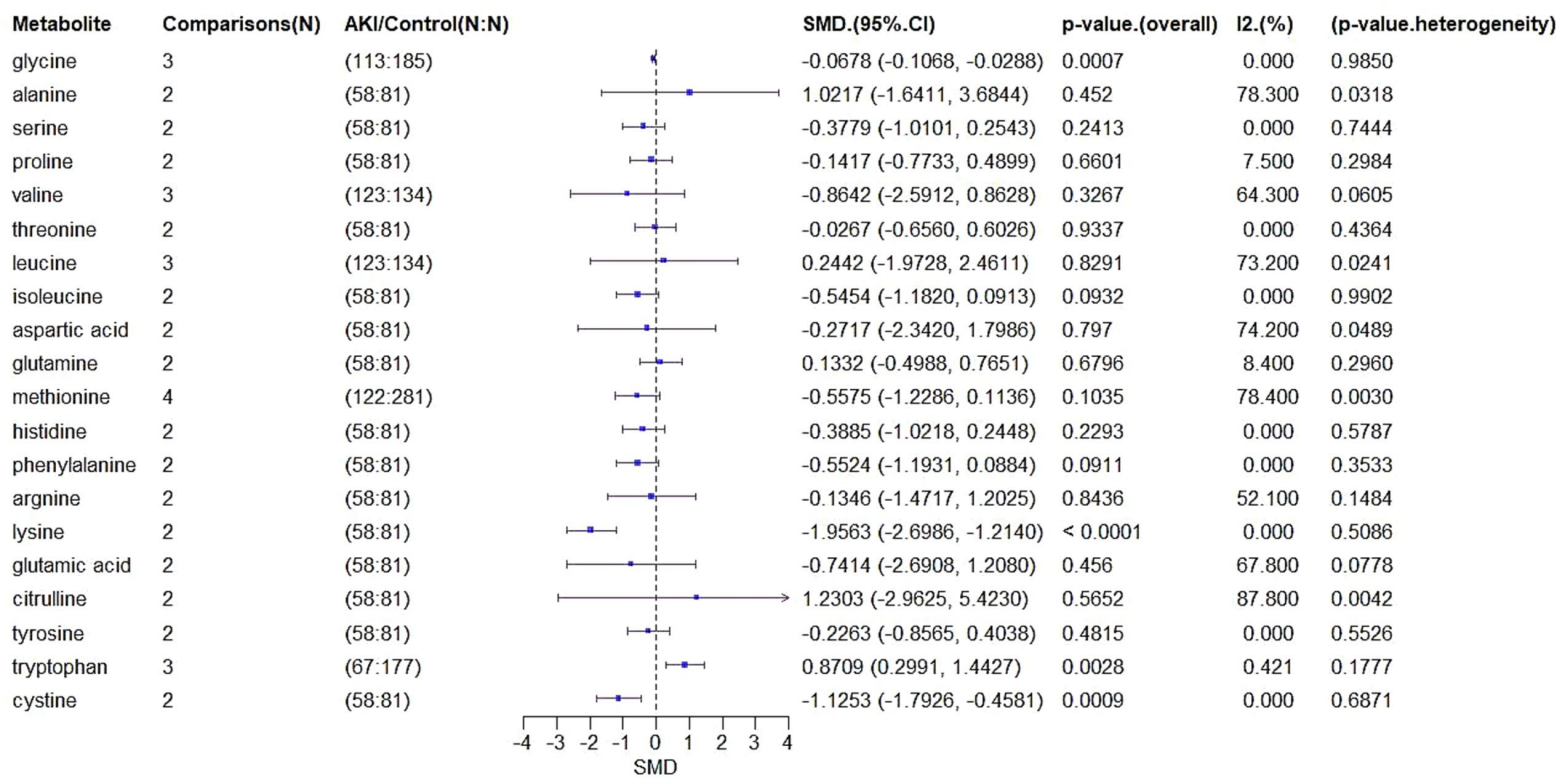
Forest plot of SMD in metabolite levels.

We extracted SMD and SE for 61 metabolites from the six studies (Figure 6). A total of 135 biomarkers were reported, and a meta-analysis was conducted on 20 metabolites that appeared across multiple studies (Table 2).

**Table 2:** Differential metabolites related to AKI in six studies.

Metabolite				
5-adenosylhomocysteine	Leucine	Histidine	Beta-alanine	N.Anserine
Glycine	Isoleucine	Phenylalanine	Aminoadipic acid	Alpha-aminobutyric acid
Serine	Aspartic acid	Argnine	Gamma-aminobutyric acid	Beta-aminoisobutyric acid
Proline	Glutamine	Citrulline	Asparagine	Carnosine
Valine	Lysine	Hippuric	Cystathionine	Ethanolamine
Threonine	Glutamic acid	Tyrosine	Homocysteine	Dehydroxylysine
Oxoproline	Methionine	Symmetric dimethyl arginine	Hydroxyproline	Methylhistidine
Citrulline	Cystathionine	Cystine	3-methylhistidine	Ornithine
O-phosphoethanolamine	O-phosphoserine	Sarcosine	Taurine	Tyrosine
Argininosuccinic acid	Homocitrulline	All-isoleucine	Serotonin	5-hydroxyindoleacetic acid
Deoxycholic acid glycine conjugate	5-Acetylamino-6-amino-3-methyluracil	L-Methionine	Tyrosyl-gamma-glutamate	Arginylarginine
2-Piperidone	3-Indolelactic acid	Leucrose	Methyl palmitate	Sophorose
Uracil				

For glycine, which was analyzed in three comparisons involving 113 AKI patients and 185 controls, the SMD was −0.0678 (95% CI: −0.1068 to −0.0288, *p* = 0.0007), indicating significantly lower levels in AKI patients. Heterogeneity was negligible (*I*^2^ = 0%, *p* = 0.985).

Alanine, based on two comparisons involving 58 AKI patients and 81 controls, showed an SMD of 1.0217 (95% CI: −1.6411 to 3.6844, *p* = 0.452), indicating no significant difference but substantial heterogeneity (*I*^2^ = 78.3%, *p* = 0.0318).

Other metabolites including serine (SMD = −0.3779, *p* = 0.2413), proline (SMD = −0.1417, *p* = 0.6601), valine (SMD = −0.8642, *p* = 0.3267), threonine (SMD = −0.0267, *p* = 0.9337), leucine (SMD = 0.2442, *p* = 0.8291), isoleucine (SMD = −0.5454, *p* = 0.0932), aspartic acid (SMD = −0.2717, *p* = 0.797), and glutamine (SMD = 0.1332, *p* = 0.6796) also showed no significant differences. However, several exhibited moderate to high heterogeneity.

Notably, lysine (SMD = −1.9563, 95% CI: −2.6986 to −1.214, *p* < 0.0001) and cystine (SMD = −1.1253, 95% CI: −1.7926 to −0.4581, *p* = 0.0009) showed significantly reduced levels in AKI patients, with no heterogeneity (*I*^2^ = 0%). Tryptophan levels increased significantly (SMD = 0.8709, 95% CI: 0.2991 to 1.4427, *p* = 0.0028, *I*^2^ = 42.1%).

These findings suggest that glycine, lysine, cysteine, and tryptophan may be candidate biomarkers for AKI, while other metabolites either showed inconsistent trends or lacked statistical significance.

### 3.5 Meta-analysis of metabolic pathway expression in AKI

**Figure 7:**
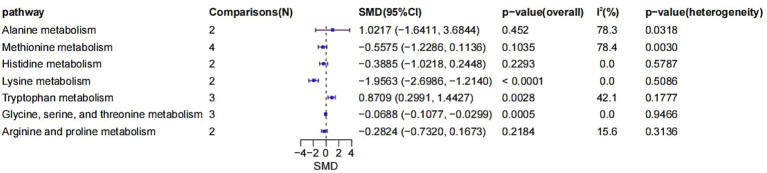
Forest plot of SMD in metabolomic pathways.

The included studies also enabled the comparison of metabolite expression across various metabolic pathways (Figure 7). First, two comparisons were included in the alanine metabolism pathway. The SMD was 1.0217 (95% CI: −1.6411 to 3.6844, *p* = 0.4520), indicating no significant difference. However, heterogeneity was high (*I*^2^ = 78.3%, *p* = 0.0318), suggesting considerable inconsistency across studies.

Four comparisons were analyzed for methionine metabolism, with an SMD of −0.5575 (95% CI: −1.2286 to 0.1136, *p* = 0.1035). Although not statistically significant, a noticeable negative trend was observed. Heterogeneity was significant (*I*^2^ = 78.4%, *p* = 0.0030).

Histidine metabolism involved two comparisons, yielding an SMD of −0.3885 (95% CI: −1.0218 to 0.2448, *p* = 0.2293), indicating no significant change. Heterogeneity was absent (*I*^2^ = 0%, *p* = 0.5787).

Lysine metabolism, based on two comparisons, showed a significantly negative SMD of −1.9563 (95% CI: −2.6986 to −1.2140, *p* < 0.0001), with no observed heterogeneity (*I*^2^ = 0%, *p* = 0.5086).

For tryptophan metabolism, three comparisons yielded a significantly positive SMD of 0.8709 (95% CI: 0.2991 to 1.4427, *p* = 0.0028), with moderate heterogeneity (*I*^2^ = 42.1%, *p* = 0.1777).

Glycine, serine, and threonine metabolism, based on three comparisons, presented a significantly negative SMD of −0.0688 (95% CI: −0.1077 to −0.0299, *p* = 0.0005), with no heterogeneity (*I*^2^ = 0%, *p* = 0.9466).

Lastly, for arginine and proline metabolism, two comparisons yielded an SMD of −0.2824 (95% CI: −0.7320 to 0.1673, *p* = 0.2184), which was not statistically significant. Heterogeneity was low (*I*^2^ = 15.6%, *p* = 0.3136).

In summary, significant negative changes were found in lysine and glycine-serine–threonine metabolism, while tryptophan metabolism demonstrated a significant positive alteration. These results suggest that specific metabolic pathways may play crucial roles in the development and progression of AKI, particularly through their involvement in oxidative stress, inflammation, and dysfunction of energy metabolism.

### 3.6 The subgroup analysis based on age revealed the possible sources of heterogeneity

We compared the effect sizes between the <60-year-old and ≥60-year-old groups using the method suggested by Cochrane. A statistically significant effect modification by age was observed for three metabolites: alanine (*Q* = 4.61, df = 1, *p* = 0.0318), aspartic acid (*Q* = 3.88, df = 1, *p* = 0.0489), and citrulline (*Q* = 8.18, df = 1, *p* = 0.0042), suggesting a more pronounced intervention-associated alteration in these metabolites among patients aged ≥60 years. No significant subgroup differences were detected for proline, leucine, arginine, or glutamine (all *p* > 0.05). Methionine was excluded from this analysis as data were available from only one study that did not report the mean age.

At the pathway level, we applied the same age-stratified subgroup analysis to several key metabolic pathways. A significant subgroup difference was identified for the alanine metabolism pathway (*Q* = 4.61, df = 1, *p* = 0.0318), providing further evidence that its response to the intervention varies by age. In contrast, a subgroup analysis for the methionine metabolism pathway was not feasible due to a lack of age-stratified data in the relevant studies.

## 4 Discussion

### 4.1 The research on the metabolomics of AKI has developed rapidly

Bibliometric analysis indicates that metabolomics studies related to AKI are gaining increasing attention, with a year-by-year rise in publication volume. However, regional disparities persist—most notably, the United States and China exhibit the most significant growth, reflecting associations with demographic changes (22) and economic development (23). Although collaborations exist among researchers and institutions, international cooperation has substantial gaps. The United States maintains strong ties with other developed countries while developing nations lag in research output and collaborative engagement. The current research focuses on (1) risk factors and management strategies, (2) clinical causes and interventions, and (3) pathophysiological mechanisms of AKI. In the early stages of disease occurrence, the body’s metabolic level can undergo sensitive changes (24). Metabolomics provides novel insights into risk factors, pathophysiological mechanisms, and therapeutic approaches (10). Biomarkers identified through metabolomics can facilitate early detection and intervention, which is helpful for precision and personalized medicine (25, 26). While some investigators have explored AKI using metabolomics, our bibliometric findings suggest that these efforts remain relatively limited. Identifying sensitive biomarkers related to AKI is not only beneficial for formulating tertiary prevention policies for community and clinical management, but also enables the development of medication regimens by revealing the pathogenesis. Therefore, based on the analysis of bibliometric data, we further explored the current limited research and employed a meta-analysis method to investigate the changes in biomarkers and metabolic pathways in depth.

### 4.2 Differences in amino acid metabolism can be used for the early diagnosis of patients with AKI

Given the kidney’s central role in amino acid metabolism, AKI can disrupt this balance (27). An important component for the body to maintain amino acid metabolic homeostasis is the nephron filtration/reabsorption mechanism occurring in the renal cortex, as well as the amino acid metabolism of renal cells (28–30). The common pathophysiological processes of AKI include endothelial dysfunction and renal tubular cell injury (31). AKI can not only affect the amino acid reabsorption process of renal tubules (32), but also damage renal cells, causing disorders in amino acid metabolism (33, 34). Therefore, Abnormal amino acid metabolism may occur in the early stage of AKI disease and be accompanied by the progression of the disease.

Our research results verify and emphasize the importance of amino acid metabolism differences in the early diagnosis of AKI. Glycine, lysine, and cystine levels were significantly decreased in AKI patients, while tryptophan levels increased, highlighting their potential as early diagnostic markers. Conversely, isoleucine approached statistical significance but may have limited diagnostic value. Currently, in clinical practice, the diagnosis and monitoring of the condition are primarily based on changes in serum creatinine levels and urine output, employing a single approach that is limited to the overall manifestations of the disease. The aforementioned metabolites show more sensitive changes and provide a more direct and intuitive reflection of the degree and source of kidney damage. Therefore, in future studies, the identified metabolites should be combined with traditional markers, such as serum creatinine and/or Neutrophil gelatinase-associated lipocalin (NGAL), to improve the sensitivity and time curves of different indicators and construct a superior integrated diagnostic model.

### 4.3 The amino acid metabolism differences in AKI patients are caused by filtration/reabsorption disorders and metabolic disorders

After amino acids are freely filtered by the glomerulus, glycine is mainly transported and reabsorbed through XT2 (SLC618) (27), while lysine (35) and cystine (36) are reabsorbed from the ultrafiltrate through rBAT/b^0,+^AT (SLC3A1/SLC7A9). The significant decrease in the levels of these three amino acids may be related to the reabsorption disorder caused by AKI. More importantly, the metabolic disorders are caused by the cell damage triggered by AKI. Serine can be converted into glycine by serine hydroxymethyltransferase in the kidneys using tetrahydrofolate as a cofactor (37). AKI can lead to a decrease in serine hydroxymethyltransferase levels, thereby blocking the production process of glycine (38). Meanwhile, metabolic acidosis induced by AKI led to an increase in the renal activity of the glycine cleavage enzyme complex, which further caused the decomposition of glycine (39, 40). Lysine is an essential amino acid that humans cannot produce. Its catabolism has two main directions, namely the saccharopine pathway and the pipecolate pathway, which primarily occur in the liver and brain, respectively (35). However, the kidneys can convert lysine into carnitine and lysine conjugates through enzymes. AKI can lead to an increase in the activity of lysine methyltransferase, causing lysine methylation to produce lysine conjugates and thereby reducing lysine levels (36). The cystine/glutamate antiporter in renal tubular cells can take up cystine and regulate intracellular glutathione synthesis to maintain cellular redox balance (41). AKI can cause an imbalance in this process and may be another reason for the decrease in cystine (42). Decreased renal function can lead to a decrease in the excretion of tryptophan and its metabolites, resulting in their accumulation in the body (27, 43). Tryptophan is an essential amino acid for humans, with 95% of it being metabolized through the kynurenine pathway. The key enzymes are indoleamine 2,3-dioxygenase (IDO) and tryptophan 2,3-dioxygenase (TDO) (44). Under normal physiological conditions, tryptophan is mainly metabolized by TDO. However, during the inflammation or stress period concurrent with AKI, the activity of TDO is inhibited, tryptophan metabolism is abnormal, and its level increases, and IDO is rapidly activated. The pathway of tryptophan metabolism shifts from being dominated by liver TDO to being dominated by extrahepatic IDO (45). Our research results emphasize the diagnostic value of amino acid metabolism alterations in AKI, which may be caused by complex disorders affecting filtration/reabsorption, as well as metabolic disorders. Further studies are needed to clarify their relationship.

### 4.4 Amino acid metabolism disorder reveals the pathogenesis of AKI

Pathway-level analysis revealed the pathogenesis of AKI. AKI caused by ischemia–reperfusion injury can induce a significant increase in lysine-specific demethylase 1 expression through the AKT pathway, thereby affecting the lysine metabolic pathway and generating oxidative stress and ferroptosis via the TLR4/NOX4 pathway (46). Another epigenetic regulatory mechanism involving lysine suggests that histone lysine crotonylation exists during AKI (47). Histone lysine crotonylation may be related to the increase in inflammatory cytokines, such as tumor necrosis factor (TNF)-like weak inducer of apoptosis (TWEAK), indicating that inflammation is also an important pathogenesis of AKI (48). Lysine supplementation, in contrast, may exert renal protective effects through diuresis, conjugate formation, and reduced albumin uptake (49).

Suppressed glycine-serine–threonine metabolism implies involvement in oxidative stress (50) and inflammation (51). Glycine can trigger the opening of the GlyR ion channel, resulting in rapid hyperpolarization, a decrease in calcium, and a reduction in the synthesis of pro-inflammatory mediators, likely via the tumor necrosis factor α (TNF-α) receptor signaling pathway (52). Glycine production and reabsorption decrease during the AKI process. Low levels of glycine can reduce the protective effect of cells as mentioned above, exacerbate inflammation, and aggravate kidney damage. The decrease in glycine levels may also have a significant impact on long-term renal function through this change in microcirculation structure (53).

The tryptophan metabolic pathway includes kynurenine and indoxyl sulfate. AKI can lead to disorders in the kynurenine pathway and indolethiol sulfate activity (54). Kynurenine and indoxyl sulfate downregulated β-catenin through the aryl hydrocarbon receptor (AHR) (55). This will lead to a decrease in microvessel density, affecting the blood flow function of the kidneys. Kynurenine can also induce immune tolerance by interfering with the development and expansion of regulatory T cells and T helper 2 through AHR (56). Furthermore, IDO, involved in the kynourine pathway, is highly expressed in antigen-presenting cells. AKI leads to the inhibition of TDO activity, and IDO is activated, which limits the proliferation and normal function of T cells (45, 56).

The decrease or increase in the metabolic pathways of these several amino acids and the occurrence and development of AKI have a sensitive and reciprocal relationship. It particularly reveals that oxidative stress, inflammation, and immune dysfunction all play a significant role in this process. This aligns with the current research hotspots, as summarized by our bibliometric analysis. This fully demonstrates that regulating these processes may have therapeutic potential.

### 4.5 The application potential and future development direction of metabolomics in the field of biomarkers for the early diagnosis of AKI

Our research summarized several sensitive amino acids that can be used for the early diagnosis of AKI. There are also some amino acids that have high heterogeneity. The high heterogeneity indicates significant differences among the studies. We attempted to explore the sources of heterogeneity through subgroup analysis and found that age might be an important factor contributing to the high heterogeneity. Some metabolites with high heterogeneity, such as alanine, showed statistically significant differences based on age. Age, gender, race, and other demographic characteristics are all directly related to kidney function status (57). We suggest that future studies should fully consider the differences in population characteristics when diagnosing and treating AKI, and establish metabolomics analyses for samples from diverse populations. Furthermore, variations in detection techniques across the original studies—such as differences in sample preparation (58), chromatography columns (59), instrument conditions (60, 61), and data processing strategies (62, 63)—have led to differences in the coverage of metabolite groups, introducing heterogeneity that may compromise the comprehensiveness and accuracy of pathway analysis. Consequently, the findings of this study primarily highlight recurrent and relatively stable metabolic pathway perturbations observed across different studies, rather than capturing the full spectrum of alterations within the metabolic network. More unified and standardized research is needed in the future to confirm the diagnostic value of these findings. Linking amino acid levels directly to immune dysfunction or ferroptosis is speculative and not directly supported by the meta-analyzed studies. However, our bibliometric data also reveal key research hotspots, providing a direction for future experimental research.

This meta-analysis only included six studies, and the available data were limited. Our analysis was limited by the inability to stratify studies by sample type, AKI etiology, or disease stage (64, 65). AKI subtypes (such as prerenal, intrinsic, and postrenal), etiologies (drugs, infections, ischemia, etc.), and progression stages are closely related (66). To enhance the adaptability and interpretability of future findings, it will be crucial to utilize larger, more diverse cohorts and conduct detailed, stratified analyses. Furthermore, a deeper investigation into the specificity and underlying mechanisms of metabolic alterations in specific pathologies is warranted.

Overall, this study advances our understanding of metabolomics in AKI and identifies key challenges. Metabolomics can comprehensively capture the functional metabolic responses of the body in a diseased state, sensitively identifying characteristic metabolic disturbances that occur before clinical observation indicators, such as decreased glomerular filtration rate and elevated serum creatinine (67, 68). Therefore, it is recommended that future research establish baseline metabolic profiles for patients with high-risk factors for AKI (such as diabetes, heart failure, old age.) and healthy controls; establish metabolic database for different etiologies of AKI (such as ischemia–reperfusion, sepsis, contrast agent.); perform dynamic metabolomics analysis at different time points of acute kidney injury (early stage, peak stage, recovery stage/transitional stage to chronic kidney disease stage), and conduct statistical correlation and integration with existing clinical observation indicators; and integrate transcriptomic and proteomic data for comprehensive biomarker mining and pathway enrichment analysis (69); and conduct a series of metabolomics monitoring before and after implementing intervention measures (such as drug treatment, nutritional support) on patients. With continued AI development, predictive models based on metabolomics and clinical features may further enhance diagnosis and treatment (70). Thus, systematic, full-course, and precise disease early diagnosis, management, and intervention can be achieved.

## Data Availability

The original contributions presented in the study are included in the article/supplementary material, further inquiries can be directed to the corresponding author.
